# Netrins and Frazzled/DCC promote the migration and mesenchymal to epithelial transition of *Drosophila* midgut cells

**DOI:** 10.1242/bio.201410827

**Published:** 2015-01-23

**Authors:** Melissa Pert, Miao Gan, Robert Saint, Michael J. Murray

**Affiliations:** 1 Department of Genetics, University of Melbourne, VIC, 3010, Australia; 2 The University of Adelaide, Adelaide, SA 5005, Australia

**Keywords:** *Drosophila*, Frazzled, Netrin, Mesenchymal epithelial transition, Midgut, Migration

## Abstract

Mesenchymal-epithelial transitions (METs) are important in both development and the growth of secondary tumours. Although the molecular basis for epithelial polarity is well studied, less is known about the cues that induce MET. Here we show that Netrins, well known as chemotropic guidance factors, provide a basal polarising cue during the *Drosophila* midgut MET. Both *netrinA* and *netrinB* are expressed in the visceral mesoderm, the substrate upon which midgut cells migrate, while their receptor *frazzled (fra)* is expressed in midgut cells. Netrins are required to polarise Fra to the basal surface, and Netrins and Fra undergo mutually-dependent endocytosis, with Fra subsequently trafficking to late endosomes. Mutations to *fra* and netrins affect both migration and MET but to different degrees. Loss of *fra* strongly delays migration, midgut cells fail to extend protrusions, and apico-basal polarisation of proteins and epithelium formation is inhibited. In netrin mutants, the migration phenotype is weaker and cells still extend protrusions. However, apico-basal polarisation of proteins, including Fra, and FActin is greatly disrupted and a monolayer fails to form. Delocalised accumulations of FActin are prevalent in netrin mutants but not *fra* mutants suggesting delocalised Fra may disrupt the MET. βPS localisation is also affected in netrin mutants in that a basal gradient is reduced while localisation to the midgut/VM interface is increased. Since a similar effect is seen when endocytosis is inhibited, Netrin and Fra may regulate Integrin turnover. The results suggest Netrin-dependent basal polarisation of Fra is critical for the formation of an epithelium.

## INTRODUCTION

Transitions between epithelial and mesenchymal cell types are an important mechanism during animal development (Thiery et al., 2009). In a mesenchymal to epithelial transition (MET), migratory mesenchymal cells organise themselves into a columnar monolayer, and establish apico-basal polarity and lateral cell-cell adhesions. METs are important in development, and in cancer progression, where they are thought to promote the growth of secondary tumours (Chaffer et al., 2007; Yao et al., 2011). The molecular mechanisms underpinning epithelial polarity have been well characterised in *Drosophila*, primarily by studies of cellularisation, the follicular epithelium, and the imaginal disc epithelia (for review see Tepass, 2012). How apico-basal polarity is initially established during an MET, however, is less well known. In some cases an existing epithelium provides instructive cues to mesenchymal cells which incorporate into the epithelium (e.g. stellate cell intercalation into the *Drosophila* malphigian tubule) (Campbell et al., 2010). For epithelia that form de-novo, contact with the extra-cellular matrix appears important. For example, in the developing *Drosophila* egg-chamber, the polarisation of the follicular epithelium begins with the establishment of a basal membrane domain on the side of the cells contacting the basement membrane, which contains βPS Integrin but excludes apical proteins such as E-Cadherin and β_Heavy_spectrin. This is prior to, and independent of apical cues associated with the germ-cell cyst (Tanentzapf et al., 2000). Similarly, when vertebrate MDCK cells form 3D epithelial cysts *in vitro*, a key initial step appears to be interaction of Integrins with the ECM, which establishes an apico-basal axis via a Rac-dependent process (O'Brien et al., 2001; Yu et al., 2005). Basal cues also appear important in the formation of the *Drosophila* midgut epithelium, the subject of this study.

The midgut forms from two mesenchymal cell masses, at opposite ends of the embryo, which migrate towards each other along the visceral mesoderm (VM). During migration the main cell type, the Primary Midgut Epithelial Cells (PMECs), progressively form an epithelium whose basal side contacts the VM. Epithelium formation depends upon contact with the VM (Tepass and Hartenstein, 1994b), and the MET is disrupted when the basally located ECM component Laminin is lacking (Yarnitzky and Volk, 1995). Whether apical cues are involved is unknown, but the key apical determinant Crumbs is not expressed and a circumferential zonula adherens belt does not form (Campbell et al., 2011; Tepass and Hartenstein, 1994a) though E-Cadherin is required for the MET (Tepass and Hartenstein, 1994b).

In a screen to find new genes regulating EMTs we identified *netrinA* (Manhire-Heath et al., 2013). Netrins are a conserved family of secreted proteins, related to the extracellular matrix proteins, laminins, with a diverse range of functions during development including axon guidance, cell migration, epithelial plasticity, and angiogenesis (reviewed in Bradford et al., 2009; Sun et al., 2011). During wing disc eversion Netrins promote the breakdown of the zonula adherens by downregulating the DCC-receptor Fra (Fra) (Manhire-Heath et al., 2013). DCC/Fra family receptors have been previously linked to epithelial adhesion and polarity. In *Drosophila*, *fra* mutant clones in eye-antennal discs cells lose epithelial polarity and appear to become invasive and migratory (VanZomeren-Dohm et al., 2011). In vertebrates, the DCC paralog Neogenin is required to maintain cell polarity and epithelial structure in the neural tube (Kee et al., 2008), and DCC promotes cell-cell adhesions in HT29 cells (Martín et al., 2006).

Given the role of DCC/Neo/Fra family receptors in epithelial morphogenesis and migration, and the fact that *netA* and *netB* are transcribed in the VM (http://www.flyexpress.net/), while *fra* is transcribed in midgut cells (Kolodziej et al., 1996), we tested for a role in the formation of the midgut epithelium. Here we show that Netrins and Fra regulate both the migration and the MET of the midgut cells, and that Fra and NetB undergo mutually dependent endocytosis. Fra plays a primary role in migration whilst Netrin polarisation of Fra to the basal membrane appears critical for the MET. In addition, we present evidence that the Netrin/Fra pathway can regulate Integrin localisation, but also show that Integrin and Frazzled pathways act in parallel to promote migration. Our findings establish Netrins and DCC receptors as new factors controlling the transition of migrating cells into an epithelium.

## MATERIALS AND METHODS

### *Drosophila* genetics

The following fly stocks were used in this study: *netAB^ΔMB23^*, *netA^Δ^*, *netB^Δ^* (Brankatschk and Dickson, 2006), *UAS-netA* and *UAS-netB* (Mitchell et al., 1996), *netAB^ΔGN^* (Newquist et al., 2013), *pebbled-GAL4* (Sweeney et al., 2007), *UAS-Fra-HA* (Garbe et al., 2007), *mys^XG43^FRT101, mys^XG43^FRT101;βν^1^, ovo^D1^FRT101;hsFLP38, ovo^D1^FRT101;hsFLP38,βν^2^* (Devenport and Brown, 2004). The following strains were obtained from the Bloomington Drosophila Stock Center: *fra^3^*, *Df(2R)BSC880*, *UAS-fra*, *UAS-YFP-rab5^DN^*, *UAS-YFP-rab5*, *UAS-GFP-Moe^ABD^*, *48Y-GAL4*, *twist-GAL4*.

Since *netA^Δ^*, *netB^Δ^* are both homozygous/hemizygous viable, all mutant embryos for these alleles were derived from homozygous/hemizygous parents. *netAB^Δ^*/Y embryos were obtained by crossing FM7/Y males to either *netAB^ΔMB23^*/FM7 female parents (hereafter *netAB^Δ^* embryos) or *netAB^ΔMB23^*/*netAB^ΔGN^* female parents (hereafter *netAB^Δ^*^(M+Z)^ embryos). Our mutant analysis of *fra* utilised the protein null allele, *fra^3^*, either homozygous or in transallelic combination with the deficiency Df(2R)BSC880.

To obtain embryos doubly mutant for *mys* and *fra*. *mys ^XG43^FRT101/ovo^D1^ FRT101*; *fra^3^/hsFLP38* females were crossed to FM7_ftz-lacZ_/Y; *fra^3^/*CyO males and embryos genotyped by immunostaining for βgal and Fra.

### Immunohistochemistry and imaging

The following primary antibodies were used: from the Developmental studies Hybridoma bank: rat anti-E-Cadherin (DCAD2, 1:100), anti-Fas3 (7G10, 1:100), anti-Fas2 (1D4, 1:100), mouse anti-β-gal (40-1a-c, 1:100), anti-beta-PS (CF.6G11, 1:20), anti-alpha-PS1(DK.1A4, 1:20), alpha-PS2(CF.2C7, 1:100); rabbit-anti-GFP (Invitrogen, 1:500), mouse-anti-GFP (Roche, 1:500), rabbit-anti-Fra (Kolodziej et al., 1996) (a gift from Florence Maschat, 1:250). Rabbit-anti-NetA a peptide antibody raised against residues 633–642 (unpublished; a gift from Ben Altenhein, 1:100), and rabbit anti-NetB (1:100)(Albrecht et al., 2011), rat anti-Cheerio (1:500) (Sokol and Cooley, 2003) (a gift from Lynn Cooley), rabbit anti-Asense (1:2000) (Brand et al., 1993) (a gift from Yuh Nung Jan), rabbit-anti-ALK (a gift from Ruth Palmer) (1:500) (Lorén et al., 2003). Secondary antibodies used were highly cross-absorbed varieties. Fluorescent Alexa-488, Alexa-568 (Invitrogen) or Dy649 (Jackson ImmunoResearch) used at 1:200.

Fluorescence microscopy was performed on an Olympus FV1000 confocal microscope. ImageJ was used for all image preparation and analysis.

### Embryonic staging and quantification of migration

Staging was based on the external morphology of the embryo, coupled with the morphology of the VM (visualised with either anti-FasIII or anti-Alk). In embryos classed as mid stage 12, the anterior lip of the germband was midway through retraction and the VM still had ∼10–30% wrapped over onto the dorsal side. At stage 13 the yolk mass viewed from the dorsal side had a rounded posterior profile and teardrop shape (not the ball like shape seen at stage 14), and the posterior end of the VM extended dorsally for a short distance, but no longer wrapped around onto the dorsal side. The gap was defined as the maximum of left and right sides normalised to the antero-posterior extent of the VM.

Germband retraction appeared normal in *netABΔ*, *fra* and *netABΔ;fra* mutant embryos and the timing of retraction in *netABΔ* and *fra^3^* mutant embryos (measured from gastrulation to mid-stage 12) was not significantly different from sibling control embryos. In contrast, some *mys* GLC, and nearly all *mys;fra* GLC embryos appeared to fail in germ retraction. For *mys;fra* embryos, therefore, all embryos were treated as stage 12.

### Quantification of βPS gradients

Confocal z-sections of the posterior midgut epithelium were taken, and an average projection representing 2.5 µm in the dorsoventral and antero-posterior axes was produced. Intensities of each antibody across this cross-section were plotted on a line graph. The VM/midgut interface was defined as the point at which the ratio of mean Alk levels on VM side versus the midgut side were at a maximum. To calculate the βPS gradient the midgut side was divided into ten sections and an average level for each section calculated to remove any fluctuations. Intensity values were normalised to the maximum and minimum of these averages. The gradient of the βPS staining was then calculated from the first to last of the five sections closest to the VM.

## RESULTS

### Netrin localisation in midgut cells is dependent on Fra

*Drosophila* contains two netrin genes, *netrinA (netA)* and *netrinB (netB)*, which lie in tandem to one another on the X chromosome (Mitchell et al., 1996). To assess the role of Netrins in midgut migration we used the alleles *netA^Δ^* and *netB^Δ^*, in which the promoter regions and first 367 and 370 residues respectively of each gene are deleted, and the small deficiency *netAB^ΔMB23^* (hereafter *netAB^Δ^*), which deletes both genes completely (Brankatschk and Dickson, 2006). We also used a recombined version of this chromosome, *netAB^ΔGN^*, which is homozygous viable (Newquist et al., 2013) (see Materials and Methods).

**Fig. 1.**
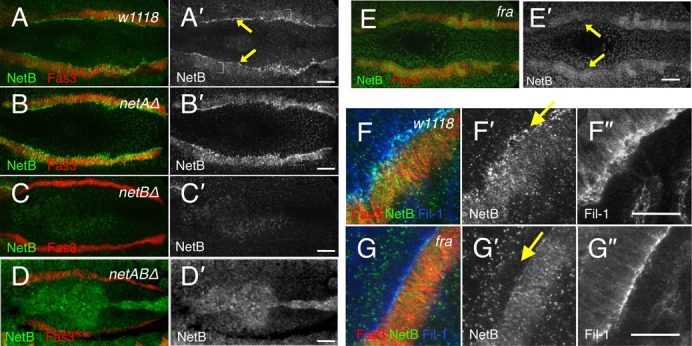
Netrin internalisation on the basal side of midgut cells requires Fra. Stage 13 embryos immunostained with Fas3 (red A–G) to identify the VM, NetB (green A–G; grey A′–G′) and Fil-1 (blue, F–G; grey F″–G″). (A) NetB is expressed in the visceral mesoderm (A′, brackets) and is basally enriched in the midgut cells (arrows) (n>50 embryos, Note: unless otherwise stated embryos depicted in figures exhibit phenotypes representative of all observed embryos). Anterior is to the left for all embryos (and throughout this paper). (B) *netA^Δ^* embryo. NetB expression appears normal (n = 18 embryos). (C) *netB^Δ^* embryo. No midgut-specific expression in observed (n = 4). (D) *netAB^Δ^* embryo. No tissue-specific expression is observed (n = 6). (E) *fra^3^/Df(2R)BSC880* embryo. NetB expression is seen in the VM, but the line of enrichment towards the basal end of midgut cells is lost (arrows) (n = 6). (F–G) High-resolution images of NetB localisation. NetB is expressed in the VM and is enriched within the midgut cells towards their basal end nearest the VM (F′, arrow). (G) *fra^3^/Df(2R)BSC880* embryo. NetB is lost from the basal side of the midgut cells (G′, arrow). Scale bars, 20 µm.

Given that *netA* and *netB* are transcribed in the visceral mesoderm, we first wished to examine distribution of Netrin proteins. The NetA antibody did not work well in embryos and showed a generally indistinct ubiquitous punctate pattern (data not shown). NetB had an embryo-wide punctate expression but was also clearly upregulated within the VM (Fig. 1A). Interestingly it was also enriched inside midgut cells, towards their basal end in both control and *netA^Δ^* embryos (Fig. 1A,B,F, arrows). In *netB^Δ^* and *netAB^Δ^* embryos both the VM-specific and midgut-specific NetB patterns were abolished (Fig. 1C,D) confirming the specificity of the staining and suggesting that there is little, if any, maternal NetB present in stage 12–13 *netAB^Δ^* embryos.

Since *netB* is transcribed in the VM we speculated that the NetB present inside midgut cells might be due to receptor mediated endocytosis. To test this we examined NetB expression in embryos lacking the receptor Fra. In these *fra* mutant embryos, NetB was still clear in the VM, but the basal puncta in midgut cells were lost (Fig. 1E,G).

Thus, Netrins, which are transcribed within the VM, becomes internalised within the adjacent midgut cells in a Fra dependent manner. Since the NetB antibody gave the clearest results the following analysis is restricted to NetB.

### Fra localisation in midgut is dependent on Netrins

**Fig. 2.**
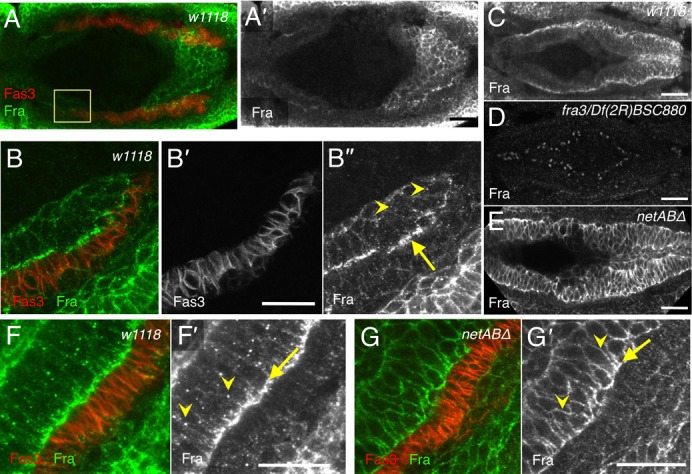
Fra basal polarisation and internal puncta are dependent upon Netrins. Stage 12 (A,B) and stage 13 (C–G) embryos stained with Fas3 (red; grey B′) and Fra (green; grey A′,B″,C–E,F′,G′). (A) Fra is expressed in the migrating midgut primordia (n = 12). (B) High-resolution image of the boxed region indicated in A. Fra expression is enhanced on the basal side of the midgut cells (B″, arrow), and in some intracellular punctae (B″, arrowheads). (C) Fra is strongly, basally polarised by stage 13, and is present in intracellular punctae (n = 13). (D) No Fra could be detected *fra^3^/Df(2R)BSC880* embryo (n = 5). (E) *netAB^Δ^* embryo. Fra expression becomes increased in the lateral membranes, and the intracellular punctate expression is lost (n = 21). (F) In a *w^1118^* embryo Fra is enriched within midgut cells towards their basal surface (F′, arrow) and localises to intracellular punctae (F′, arrowheads). (G) In a *netAB^Δ^* embryo basal polarisation is less pronounced (arrow), levels on lateral membranes are increased (arrowheads) and punctae are lost. Scale bars, 20 µm.

In stage 12 control embryos, Fra localised to the plasma membrane, and was enriched on the basal side of the migrating midgut cells (Fig. 2A,B). In the cytoplasm Fra appeared speckled with large, conspicuous puncta evident (Fig. 2B″, arrowheads). At stage 13 the basal enrichment and strong intracellular punctate staining were also clear especially in the cells towards the posterior of the embryo (Fig. 2C,F). No midgut-specific expression pattern was observed in *fra* mutant embryos (Fig. 2D), confirming the specificity of the antibody and that there is little, if any, maternal Fra protein in stage 13 *fra* mutant embryos. In *netAB^Δ^* mutants the basal polarisation of Fra was less pronounced, while expression along the lateral membranes was increased (Fig. 2E,G). Furthermore no intracellular Fra punctae were observed suggesting that Fra requires Netrins for internalisation.

**Fig. 3.**
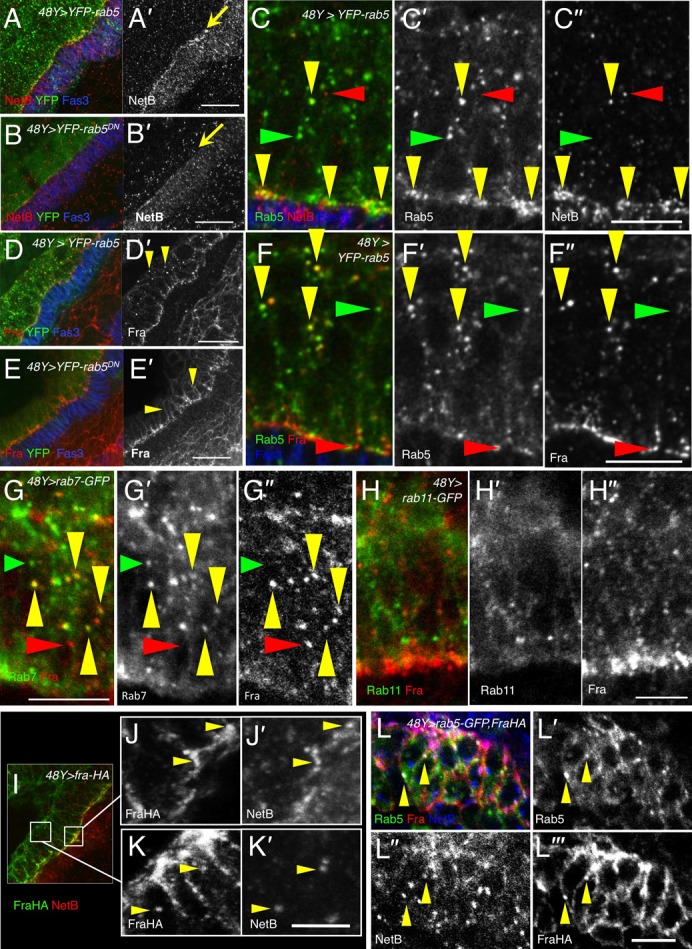
NetB and Fra endocytosis and colocalisation. Stage 13 embryos immunostained for Fas3 (blue A–F), NetB (red A–C,I; blue L; grey A′,B′,C″,J′,K′,L″), Fra (red, D–H; grey D′,E′,F″–H″), GFP (green, A–H,L; grey C′,F′,G′,H′,L′) and HA (green I; grey K,L″′). (A) In embryos expressing YFP-Rab5 (green), NetB (red) is enriched in the basal regions of the midgut (arrow) (n = 5). (B) This line is greatly reduced in embryos expressing dominant-negative *YFP-rab5^S43N^* (B′, arrow) (n = 8). (C) NetB colocalises with YFP-Rab5 extensively in basal regions of the cell, and in some internal puncta (yellow arrowheads) (n = 5). (*Note: some puncta express only NetB/Fra (red arrowheads), while others express only Rabs (green arrowheads) indicating that colocalisation in C, F and G is not due to “bleedthrough” between channels*). (D) In embryos expressing YFP-Rab5 (green), Fra (red) is enriched in the basal regions of the midgut and in many internal puncta (arrowheads) (n = 8). (E) In embryos expressing dominant-negative *YFP-rab5^S43N^* internal puncta are absent and stronger lateral membrane staining is seen (E′, arrow) (n = 5). (F) Fra colocalises with Rab5 in many internal puncta (yellow arrowheads) (n = 8). (G) Fra colocalises with Rab7-GFP expressing late endosomes (n = 5). (H) No colocalisation was seen with the recycling endosomal marker Rab11-GFP (n = 7). (I–K) Co-localisation between NetB and Fra. A stage 13 embryo *48Y-GAL4/+;UAS-fra-HA/+* embryo, stained with HA (green) to show Fra expression and NetB (red) (n = 4). (I) Co-localisation between NetB and Fra was seen predominately on the basal side of the midgut cells. (J–J′). Zoomed image of the region indicated by the right box in I. Arrowheads indicate co-localisation between NetB and Fra on the basal side of the midgut. (K–K′) Zoomed image of the left box in I. NetB and Fra-HA colocalise in some internal punctae. (L) Stage 13 embryo co-expressing Rab5-GFP and Fra-HA shows colocalisation of NetB, Rab5 and Fra in some internal puncta (arrowheads) (n = 5). Scale bars, 20 µm (A,B,D,E); 10 µm (C,F,G,H,J,K,L).

In vertebrate neurons, Netrin-1 can induce endocytosis (Piper et al., 2005) and downregulation (Kim et al., 2005) of its receptor DCC, a mechanism thought to allow growing axons to become desensitised to their guidance cues. Since a similar system might be operating in the midgut we therefore wished to determine whether Netrins were inducing endocytosis of Fra in midgut cells. We first tested whether the NetB and Fra punctae colocalised with the endosome marker YFP-Rab5 (Zhang et al., 2007). Rab5 is present on both the plasma membrane and in early endosomes (Chavrier et al., 1991), and is required in the fusion of plasma membrane-derived endocytic vesicles with early endosomes (Bucci et al., 1992). Some co-localisation between YFP-Rab5 positive endosomes and NetB punctae was seen along the basal side of the midgut (Fig. 3A,C). Co-localisation of YFP-Rab5 with Fra was also seen in the basal parts of midgut cells, but also in more apically situated punctae (Fig. 3D,F). Inhibition of endocytosis by expression of the dominant negative transgene *UAS-YFP-rab5^S43N^* altered both NetB and Fra expression patterns. NetB enrichment in the basal regions of the midgut cells was greatly reduced (Fig. 3B), suggesting that endocytosis of NetB on the basal side of the midgut was being blocked. Similarly, although Fra was still basally polarised, the intracellular punctae were lost and more Fra was found on lateral membranes (Fig. 3E). We also tested Fra colocalisation with the late endosome marker Rab7-GFP and the recycling endosome marker Rab11-GFP. Fra extensively colocalised with Rab7-GFP (Fig. 3G), but not Rab11-GFP (Fig. 3H), suggesting that endocytosed Fra may be targeted for degradation, as in axons.

To test for colocalisation of NetB and Fra we expressed a HA-tagged *fra* transgene in the midgut. Fra-HA largely recapitulated the endogenous Fra localisation with internal punctae and basal polarisation, though there was more localisation to cell membranes perhaps due to higher expression levels. Fra-HA and NetB co-localised on the basal surface of the midgut (Fig. 3I,J) and in 36.9%±2.3 (SEM, n = 5) of the intracellular, FraHA-positive punctae (Fig. 3K) (see supplementary material Fig. S1). Both Fra-HA and NetB colocalised with Rab5 (Fig. 3L).

Taken together the results suggest that NetB and Fra are endocytosed together, in a mutually dependent manner, at the basal surface of the midgut.

### Netrins and Fra are required for embryonic midgut migration

**Fig. 4.**
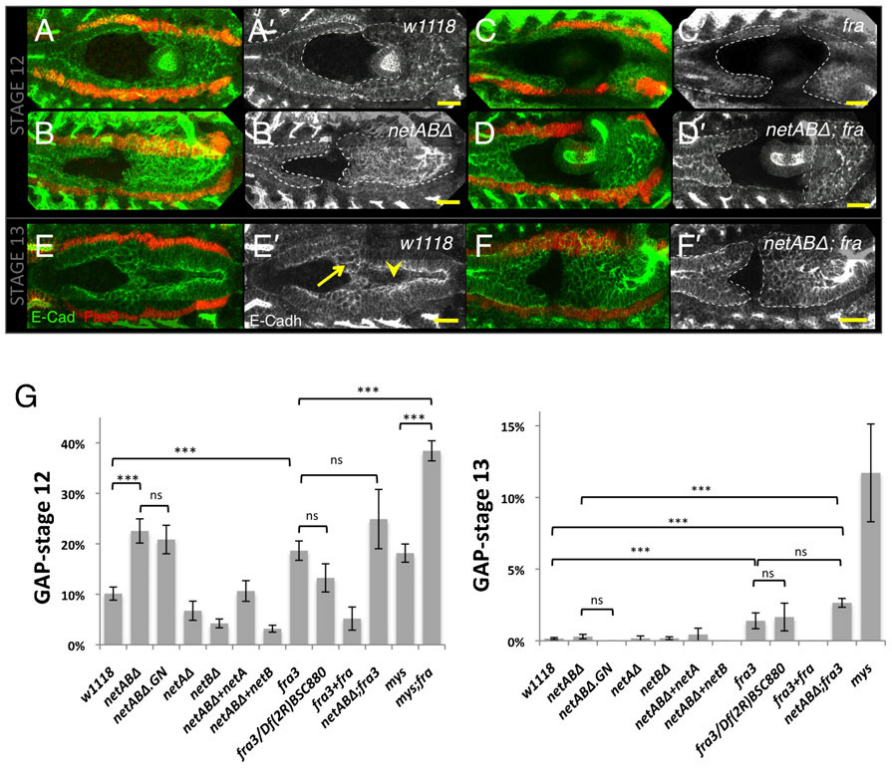
Embryonic midgut migration is delayed in netrin and *fra* mutants. (A–F) Stage 12 (A–D) and stage 13 (E–F) embryos immunostained for Fas3 (red) to identify the VM, and for E-Cadherin (green; grey A′–F′) to identify the midgut. Dotted line depicts extent of midgut. (A) *w^1118^* control embryo with the midgut primordia just meeting. (B) In a *netAB^Δ^* embryo migration is delayed. (C) *fra^3^/Df(2R)BSC880* mutant embryo showing a greater migration delay. (D) Combined loss of netrins and *fra* enhanced the delay phenotype. (E) Stage 13 *w^1118^* control embryo. The epithelium has formed, and only ICPs (arrow) and AMPs (arrowhead) are yet to incorporate into the epithelium. (F) *netAB^Δ^;fra^3^* mutant. A gap between the primordia is still evident. Note: The VM was well formed and continuous in all of these genotypes. Any apparent breaks are due to the VM being outside the focal planes shown. (G) Quantification of migration delay in stage 12 and stage 13 embryos. For p-values and n-values see Table 1. Scale bars, 20 µm. ***  =  p<0.001, ns  =  p>0.05.

We next determined if Netrins and Fra played a functional role in midgut migration. To quantify migration rates we stained embryos for either Filamin-1 (Cheerio) or E-Cadherin to visualise the midgut cells and measured the maximum gap between anterior and posterior midgut rudiments, as a fraction of total VM length, at two key stages: mid stage 12, when cells in wild type embryos are just meeting and stage 13, when migration is complete (see Materials and Methods for staging criteria). Migration was not affected in *netA^Δ^* embryos and, surprisingly, was slightly accelerated in *netB^Δ^* embryos (Fig. 4G; Table 1). Migration was significantly delayed in *netAB^Δ^/Y* (hereafter *netAB^Δ^*) embryos at stage 12 (Fig. 4B), though the posterior and anterior midgut rudiments had met by stage 13 (Fig. 4G; Table 1). In *netAB^Δ^* (and *fra* mutants) the VM appeared normally formed and expressed the usual VM markers, Fas3 and Alk. The *netAB^Δ^* migration delay was rescued by mesoderm-specific expression of either *netA* or *netB* using the *twist-GAL4* driver (Fig. 4G; Table 1). Since migration only showed a delay when both netrins were deleted, and either gene could rescue that delay, we conclude that the two paralogs act redundantly in midgut migration.

**Table 1.**
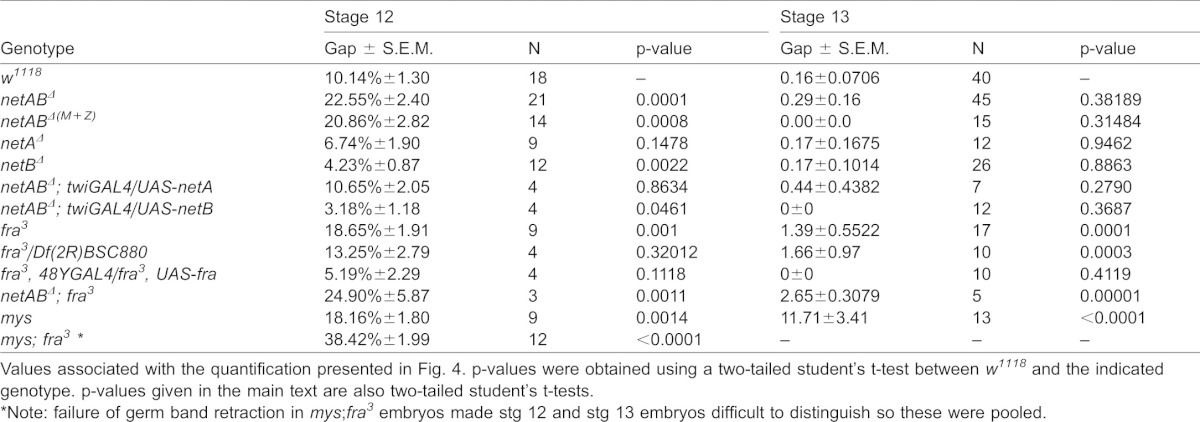
Midgut migration gap at stage 12 and 13

To assess migration in embryos lacking Fra we examined embryos homozygous for the protein null allele, *fra^3^*, or heterozygous for *fra^3^* and the deficiency *Df(2R)BSC880*, which deletes *fra*. Migration rates for these genotypes were not significantly different (p = 0.14 at stage 12, p = 0.8 at stage 13), but were delayed in comparison with control embryos at stage 12 (Fig. 4C,G; Table 1). This delay was comparable to *netAB^Δ^* mutants at stage 12, but by stage 13 *fra* mutants still exhibited a gap (unlike *netAB^Δ^* mutants) (Fig. 4G), though this was closed at later stages (data not shown). The delay was rescued by expression of a *fra* transgene using the midgut driver *48Y-GAL4* (Martin-Bermudo et al., 1997) (Fig. 4G; Table 1). Since migration in *fra* mutants was more strongly affected than in *netABΔ* mutant we speculated that the zygotic null *netABΔ* embryos might be being partly rescued by maternal Netrin. However, migration rates in *netAB^Δ^* ^(M+Z)^ embryos (which are both maternal and zygotic null - see Materials and Methods) were not significantly different from *netABΔ* embryos at stage 12.5 (p = 0.65) or stage 13 (p = 0.30) (Fig. 4G). This implies that Fra has some Netrin-independent activity in migration. Combined loss of netrins and *fra* enhanced the *fra* phenotype, though the difference was not significant, with an increased delay at stage 12 (p = 0.2), and a larger gap remaining at stage 13 (p = 0.24) (Fig. 4D,F,G; Table 1). As with *fra* and *netAB^Δ^* mutants the gap was also closed at later stages (data not shown).

**Fig. 5.**
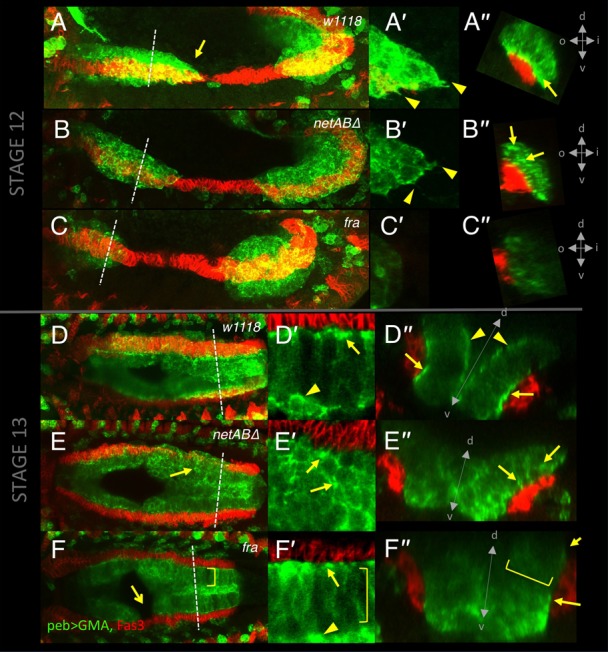
FActin distribution and cellular morphology in control, netrin and *fra* mutant embryos. Stage 12 (A–C) and 13 (D–F) embryos immunostained for Fas3 (red) and GFP (green) in which the FActin marker GFP-Moe^ABD^ is expressed by the *pebbled-GAL4* driver, in control (A,D), *netAB^Δ^* (B,E) and *fra^3^/Df(2R)BSC880* (C,F) mutant embryos. A′–C′ show the migrating front of the anterior midgut. A″–F″ show cross-sections at dotted lines in A–F. D′–F′ show a magnified image of the nascent epithelium in the posterior half of the midgut. (A) At mid stage 12 midgut cells from the anterior and posterior primordia are moving together (n = 8). Cells form a streamlined wedge shape (arrow), and extend protrusions (A′, arrowheads). FActin is enriched basally at the point of contact with the VM (A″, arrow). (B) In *netAB^Δ^* mutants the overall shape of the migrating anterior midgut primordium is similar, and protrusions are evident (B′). Patches of FActin enrichment are present (arrows) but are not polarised to the basal side (B″) (n = 19). (C) In *fra* mutants, the midgut has a smoother profile (C′), basal polarisation is not clear, and patches of FActin enrichments are less obvious (C″) (n = 6). (D) Control embryo at stage 13. The midgut cells have organised themselves into a columnar monolayer. Due to variable GAL4 expression levels individual cells can be distinguished, extending from the VM through to the AMP cells on the apical surface of the epithelium (arrowhead). FActin is still basally polarised (D′,D″, arrows), though there is also some enrichment at the apical surface (D″, arrowhead) (n = 5). (E) In *netAB^Δ^* mutants the columnar arrangement is less apparent (E′). FActin patches are prevalent (arrows) but located around cell bodies, and not polarised to the basal surface (E″) (n = 11). (F) *fra* mutant, in which a gap is still evident (arrow). The epithelium is closer to wild type (F′), and basal polarisation of FActin is greatly reduced (F″, arrow) (n = 5). FActin patches seen on lateral membranes in *netAB^Δ^* embryos are missing (F,F′,F″ brackets). A″–F″ (d = dorsal, v = ventral, i = inside, o = outside).

Next we examined the morphology of migrating cells with the FActin reporter GFP-Moe^ABD^ (GMA) (Kiehart et al., 2000). At mid-stage 12 the cells at the front in control embryos were flattened out upon the VM in the direction of motion, giving the mass of cells a wedge shape (Fig. 5A). Cells at the front extended fine protrusions (Fig. 5A′) and the FActin was concentrated at the point of contact with the VM (Fig. 5A″). In *netAB^Δ^* mutants the streamlined shape and protrusions were present though less prominent than in controls (Fig. 5B,B′). Patches of strong FActin accumulation were still present but no longer polarised to the basal surface (Fig. 5B″). Similar results were seen for *netABΔ^(M+Z)^* mutants (data not shown). In *fra* mutants the midgut primordia were more rounded, protrusions were rare, and the enrichment of FActin at the basal contact point was greatly reduced (Fig. 5C). Thus the formation of protrusions and FActin accumulations, and the stretching out of PMECs along the VM occur more normally in *netAB^Δ^* mutants than in *fra* mutants again suggesting Fra has some netrin-independent functionality.

### Netrins and Fra are required for the formation of a columnar epithelium

**Fig. 6.**
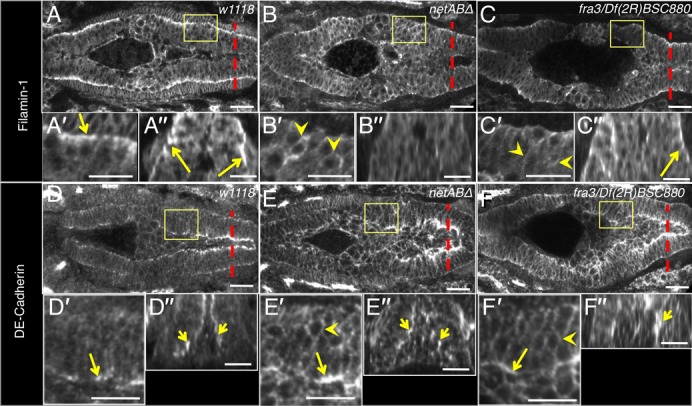
Netrins and Fra are required for the midgut MET. Stage 13 embryos showing disruption of epithelium formation. Boxed regions in (A–F) are magnified in (A′–F′), and cross-sections taken at the dotted lines are shown in (A″–F″). (A) *w^1118^* control embryo. Filamin-1 is basally polarised (arrows; A′–A″) (n = 27). (B) *netAB^Δ^* embryo. Basal polarisation of Filamin-1 is lacking (B′–B″). Instead it is distributed around the entire cell membranes (arrowheads) (n = 28). (C) *fra^3^/Df(2R)BSC880* embryo. Basal polarisation is reduced though not absent (C″, arrow) and expression is increased around the entire cell membranes (arrowheads) (n = 14*). (D) *w^1118^* embryo. E-Cadherin is apically polarised in the midgut cells (arrows) (n = 10). (E,F) In *netAB^Δ^* embryo (n = 9) and *fra^3^/Df(2R)BSC880* embryos (n = 10) E-Cadherin apical localisation is reduced but still apparent (arrows) and shows increased expression around the entire cell membranes (arrowheads). * for fra *mutants, n-values are pooled from* fra^3^*/Df(2R)BSC880 and* fra^3^*/*fra^3^ *genotypes which exhibited the same phenotype*. Scale bars, 20 µm.

Next we tested whether the formation of the epithelium was affected in *netAB^Δ^* and *fra* mutants. At stage 13, following completion of migration, the PMECs form a columnar epithelium with apico-basal polarisation of proteins, such as Filamin-1 (Fig. 6A). In *netAB^Δ^* mutants Filamin-1 was more evenly distributed around the cells, and cells were more rounded and were not organised into a single layer (Fig. 6B). A similar though less severe defect was seen in *fra* mutants (Fig. 6C). The basal enrichment of FActin seen during migration was also a feature of the nascent epithelium in control embryos (Fig. 5D″, arrows). In *netAB^Δ^* mutants FActin accumulations were prevalent around the cell membranes but were not polarised to the basal side (Fig. 5E″). In *fra* mutants there was a small amount of FActin at the basal side but a lack of FActin accumulations in the lateral parts of the cells (Fig. 5F″). Localisation of E-Cadherin was also affected. E-Cadherin is basally localised during migration at stage 12 (data not shown), but becomes enriched on the apical surface at stage 13 (Fig. 6D). In *netAB^Δ^* and *fra* mutants, E-Cadherin expression was disrupted, with increased expression around the entire cell membranes (Fig. 6E,F). The failure in the formation of a columnar epithelial layer in *netABΔ* embryos was clearest in embryos stained for the cell-cell adhesion molecule Fas2, which marks the shared membranes between adjacent PMECs (supplementary material Movies 1, 2).

The effects on FActin accumulations in *netAB^Δ^* and fra mutants is similar to a recent report concerning the worm orthologues UNC-6 (Netrin) and UNC-40 (Fra) in the anchor cell of the worm, *C. elegans*. FActin clusters are normally enriched at the basal membrane, but in *unc-6* mutants undergoes repeated cycles of accumulation and dissolution at random locations around the cell, whilst in *unc-40* mutants these strong accumulations are missing and FActin is weakly polarised to the basal surface (Wang et al., 2014). Thus, the failure to undergo the MET correlates with mislocalised accumulation of both Fra and FActin. These results establish the Netrin pathway as a new regulator of the midgut MET.

### Adult midgut precursors are correctly specified but misplaced in netrin mutants

**Fig. 7.**
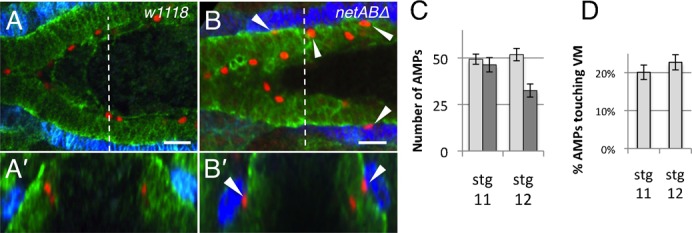
Adult midgut precursor cells are mislocalised in *netAB^Δ^* mutant embryos. (A,B) Stage 12 embryos immunostained with Fas3 (blue) to mark the VM, Filamin-1 (green) to mark the midgut and Asense (red) to mark the Adult Midgut Precursor (AMP) cells. Images show only the anterior midgut. (A′–B′) represent cross sections taken at the dotted line in (A,B). (A) The AMPs in *w^1118^* embryos are located on the apical surface of the developing midgut epithelium. None come into contact with the VM. (B) In *netAB^Δ^* embryos, some AMPs are found in contact with the VM (arrowheads). (C) Quantification of AMP numbers in *w^1118^* (light grey) (n = 3, n = 5, at stg 11, 12 resp.) and *netAB^Δ^* embryos (n = 3, n = 4, at stage 11, 12 resp.). (D) Proportion of AMPs in contact with the VM in *netAB^Δ^* embryos. Scale bars, 20 µm.

In addition to the PMECs, the midgut consists of two other cell types, the Adult Midgut Precursors (AMPs) and the Interstitial Cell Precursors (ICPs). AMPs and ICPs, which express the neural precursor gene *asense* (Tepass and Hartenstein, 1995), maintain a more mesenchymal phenotype and only incorporate into the epithelium at later stages (Tepass and Hartenstein, 1995). Since cell fate changes, in which PMECs are transformed towards the AMP fate, can disrupt migration (Tepass and Hartenstein, 1995) we also checked if the cell populations were normally specified in netrin mutants. Stage 11 and 12 embryos immunostained for Asense showed the expected population of AMPs and ICPs, though the position and number of AMPs was altered. In control embryos we were able to detect ∼50 AMPs in the anterior half of the embryo (7C) (Fig. 7A; n = 8 embryos). In *netAB^Δ^* embryos, there were only ∼30–40 AMPs detectable (Fig. 7B,C; n = 7 embryos). Since migration is delayed in *netABΔ* embryos it may be that some AMPs had not yet migrated out of the head regions and were therefore not accounted for. In control embryos all AMPs were positioned on the apical surface of the PMEC cells, whereas in *netABΔ* embryos ∼20% of AMPs were in contact with the VM (Fig. 7B, arrowheads, Fig. 7D), a highly significant difference (p<0.001 at both stage 11 and 12). We speculate that the early intercalation phenotype is due to the failure in the formation of an epithelium, i.e. normally the strong lateral cell-cell and basal cell-ECM adhesions of the midgut epithelium inhibit AMP intercalation until later stages.

### Netrin/Fra internalisation is not dependent on Integrins

We next wished to understand how the Netrin/Fra pathway might relate to the other well established signalling pathway regulating midgut development, the Integrins. These two molecular pathways could have significant cross-talk since several signalling components (e.g. FAK) are known to act downstream of both DCC family receptors and Integrins. In addition, direct binding between Netrins and an Integrin has been demonstrated using human pancreatic epithelial cells, which could adhere to and migrate upon Netrin-1 *in vitro* via the α6β4 Integrin receptor (Yebra et al., 2003).

We first tested whether Netrins, Fra and Integrins were dependent upon each other for correct localisation. In *Drosophila* there are two β Integrin subunits, βPS and βν, and five α subunits, αPS1/mew, αPS2/if, αPS3/scb, αPS4, αPS5 (Brown, 2000; Devenport and Brown, 2004). βPS, is expressed widely in the embryo while βν is specific to the midgut. Germline clones of βPS (hereafter βPS mutants) show delays in midgut migration, while in mutants lacking both βPS and βν (i.e. complete Integrins nulls) migration completely fails (Devenport and Brown, 2004).

**Fig. 8.**
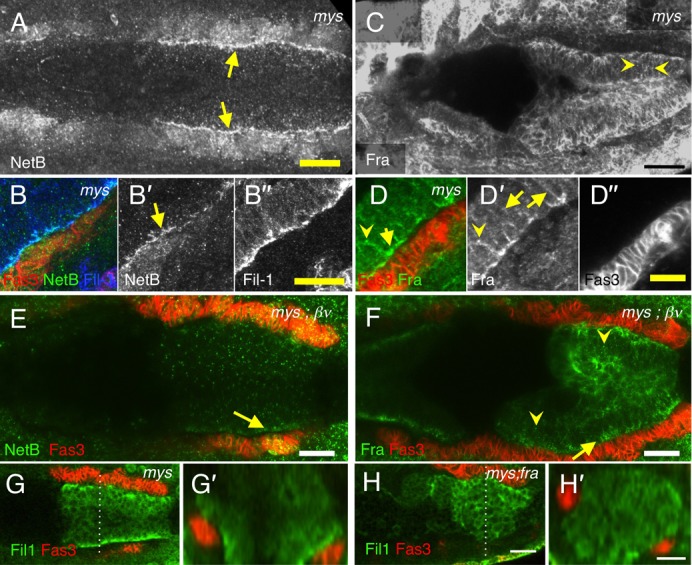
NetB and Fra expression in integrin mutants. *mys* (A–D) and *mys;βν* (E,F) maternal/zygotic mutants stained with Fas3 (red B,D–H; grey D″), Fil-1 (blue B; green G,H; grey B″), NetB (green B,E; grey A,B′), and Fra (green D,F; grey C,D′). (A,B) In stage 13 *mys* embryos NetB expression appears normal, with a strong line of enrichment, basally in the midgut cells (arrows). Note that Filamin-1 also shows normal basal polarisation (B″) as previously reported (Devenport and Brown, 2004). (n = 4) (C,D) In stage 13 *mys* mutant embryos Fra expression is relatively normal with enrichment on the basal side of the midgut (C,D′) and intracellular punctae (arrowheads), though localisation to lateral membranes appeared slightly stronger (arrows). (n = 5) (E) *mys;βν* embryo showing basal enrichment of NetB within midgut cells (E′, arrow). (n = 5) (F) *mys;βν* embryo (stage 13) showing Fra basal enrichment (arrow) and intracellular punctae (arrowheads) (n = 5). (G–H) Basal polarisation of Filamin-1 is clear in stage 12 and 13 *mys* embryos (G) (n = 13) but lost in *mys;fra^3^* mutant embryos (n = 12). G′ and H′ show cross sections of G and H at the position of the dotted lines. Scale bars, 20 µm (A,C,E,F,G,H); 10 µm (B,D).

In *βPS* mutants, NetB was still expressed in the VM and basally enriched in the midgut cells (Fig. 8A,B; compare to Fig. 1A,F). Basal polarisation of Filamin-1 was normal (Fig. 8B″) as previously reported (Devenport and Brown, 2004). Fra was also basally polarised and punctate as in control embryos (Fig. 8C,D; compare to Fig. 2C,F) though there appeared to be a modest increase in expression at the lateral membranes (arrows in Fig. 8D′). In embryos lacking both βPS and βν midgut development was highly disrupted making it difficult to assess whether NetB and Fra localisations were completely normal. Nevertheless, in embryos that appeared to be approximately stage 13 one could still clearly see basal enrichment and internal puncta for NetB (Fig. 8E) and Fra in midgut cells (Fig. 8F).

The results show that the key features of NetB and Fra localisation, polarisation and internalisation, are not dependent upon Integrins though, Integrins may play a subtler role in regulating Fra given the redistribution to the lateral membrane.

### Integrin βPS/aPS1 localisation is dependent upon Netrin/Fra signalling

**Fig. 9.**
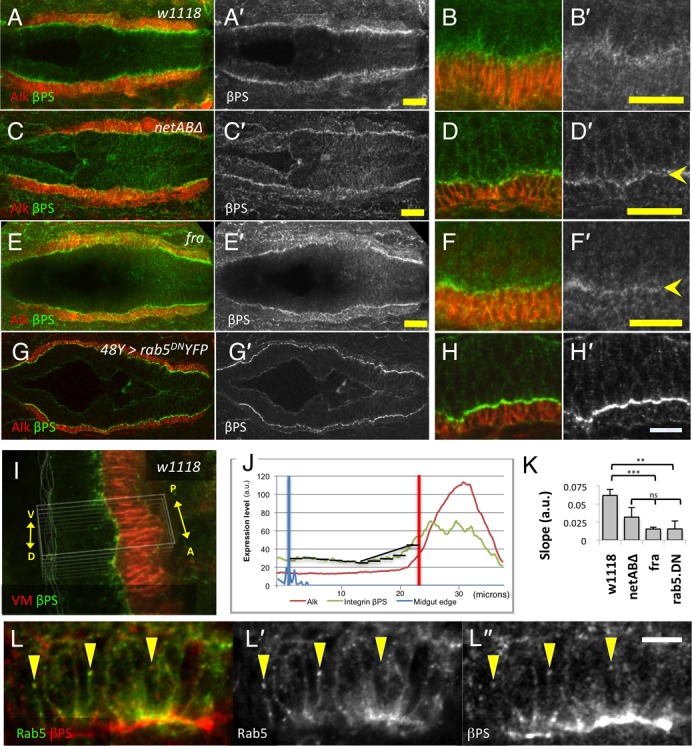
Integrin expression is altered in netrin and *fra* mutant embryos. Stage 13 embryos stained with Alk (red) to mark the VM and Integrin βPS (green A–H; grey A′–H′). Embryos in A,C,E and G are shown at higher resolution in B,D,F,H. (A,B) *w^1118^* embryo. βPS is expressed in the VM and the midgut. Midgut expression is strongest on the basal side of the midgut cells, and forms a gradient going apically into the cell. (C–F) *netAB^Δ^* (C,D) and *fra^3^/Df(2R)BSC880* (E,F) embryos. The βPS gradient of expression in the midgut is greatly reduced, and a more distinct line of βPS expression is seen at the interface between the VM and midgut (D′,F′, arrowheads). (G,H) Similar changes are seen in embryos in which endocytosis is inhibited by expression of dominant negative YFP-Rab5. Arrowhead in H′ indicates increased expression on lateral membranes. (I,J) Method of quantifying βPS gradient. A small section of the midgut epithelium was averaged in both the anterior-posterior and dorso-ventral directions to arrive at a single intensity profile for βPS (green) and Alk (red) (J). The normalised βPS slope for the basal half of the midgut was determined (see Methods for details). (K) Mean slopes for the four genotypes. N-values, and p-values with respect to *w^1118^* (student's t-test) are *w^1118^* (n = 6), *netAB^Δ^* (n = 6, p = 0.082), *fra* (n = 6, p<0.001) and *rab5^DN^* (n = 5, p<0.01). The three mutant genotypes were not significantly different from each other. (L) Colocalisation of the early endosome marker Rab5-GFP (green L; grey L′) and βPS (red L; grey L″) (arrowheads). Note exposure level for βPS is much higher than in panels A–H. Scale bars, 20 µm in A,C,E; 10 µm in B,D,F,H,L. ***  =  p<0.001, ns  =  p>0.05.

Next we tested whether localisation of Integrins was dependent upon Netrins or Fra. We examined localisation of βPS, which is expressed in both midgut and VM cells, αPS1, which is expressed in the midgut cells, and αPS2, which is expressed in the VM (Bogaert et al., 1987; Leptin et al., 1989; Wehrli et al., 1993). In stage 13 control embryos, βPS was expressed throughout the VM and in the midgut and was punctate. In the midgut, an apico-basal gradient of expression was observed with highest levels towards the basal regions of the midgut cells (Fig. 9A,B; For quantification methods and results see Fig. 9I–K and Materials and Methods). In both *netAB^Δ^* (Fig. 9C,D) and *fra* (Fig. 9E,F) embryos βPS was present within midgut cells but did not form an obvious gradient (quantified in Fig. 9K). Instead an increased line of expression was apparent at the interface between the midgut and VM (Fig. 9D,F, arrowheads).

Immunostaining for αPS1, which is known to form heterodimers with βPS in the midgut, did not work well in control embryos and exhibited no obvious pattern. Interestingly, however, like βPS, a weak line of αPS1 was observed at the interface between the midgut and VM in *netAB^Δ^* embryos, and even more clearly in *fra* embryos, though never in controls (supplementary material Fig. S2). This suggests that loss of Net/Fra signaling somehow changes the levels of αPS1 at the plasma membrane or its accessibility to antibodies.

Finally, αPS2, which was clearly expressed in the VM and also localised to the midgut/VM interface was not affected in netrin and *fra* mutants (data not shown). Similarly localisation of the ECM components Nidogen and Laminin B appeared normal suggesting that changes in Integrin localisation were not due to gross changes to the ECM (data not shown).

Integrins are known to undergo endocytosis and recycling (Margadant et al., 2011), and internalisation of Integrins has been documented in *Drosophila* (Yuan et al., 2010). We speculated therefore that the βPS gradient might be due to endocytic turnover of Integrins on the basal surface of midgut cells, and that the loss of a gradient and increased levels of αPS1/βPS at the plasma membrane in *netAB^Δ^* and *fra* mutants might be due to a disruption of this turnover. We therefore examined whether βPS colocalised with Rab5, and whether it was affected by inhibition of endocytosis with Rab5^DN^. βPS did extensively colocalise with Rab5 (Fig. 9L). In addition, expression of *UAS-YFP-rab5^S43N^* had a similar, though more pronounced, effect on the localisation of βPS to loss of netrins or *fra*, with a reduction in the gradient and increased localisation to the PMEC/VM interface (Fig. 9G,H,K).

Since this raised the possibility that migration defects in *fra* mutants could potentially be due to some effect on Integrins, we tested the effects of combined loss of *fra* and *mys*. We compared migration rates, and Filamin-1 polarisation in *mys* germline clones with and without Fra. Migration in *mys;fra* embryos was clearly more delayed than in either *mys* or *fra* alone (p<0.001) (Fig. 4G), and as expected the normal polarisation of Filamin-1 apparent in *mys* embryos (Fig. 8G) was lost in *mys;fra* double mutants (Fig. 8H).

Thus, localisation of βPS/αPS1, is dependent upon Netrin/Fra pathways, which may promote Integrin turnover at the basal plasma membrane. However, it is clear that Integrins and Frazzled play independent, additive roles in migration.

## DISCUSSION

### Netrins and Fra are required for midgut migration

We have shown that Netrins and Fra are important both for the migration of midgut cells and for their transition into a polarised, monolayered epithelium. Their role in migration appears to be a typical example of chemoattraction whereby cells/axons expressing a receptor follow a pathway that expresses the ligand. Fra is critical for this role, since the fine protrusions, strong FActin accumulations and wedge-shaped morphology of midgut cells were absent in *fra* mutants and migration was strongly affected. *netAB^Δ^* mutants, both zygotic and maternal+zygotic nulls, exhibited a similar but less pronounced migration delay. NetA and NetB play redundant roles in this migration since a delay only occurred when both genes were deleted, and either gene could rescue that delay. These results also imply that Fra can activate motility signaling pathways in the absence of Netrins. A similar effect has been seen in *C. elegans* where the Fra orthologue UNC-40 has several roles that are independent of the netrin orthologue UNC-6 (Alexander et al., 2009; Honigberg and Kenyon, 2000; Yu et al., 2002) (and see Discussion below).

### Netrins and Fra are required for the midgut MET

Loss of Netrins, and to a lesser extent Fra, also disrupted apico-basal polarisation of Filamin-1, FActin and E-Cadherin, and the formation of a columnar, monolayered epithelium. In addition, in *netAB^Δ^* mutants, AMP cells, were able to intercalate into the PMEC layer by stage 12, an event that doesn't usually occur until stage 14, when the columnar epithelium, which is dependent upon E-Cadherin adhesion (Tepass and Hartenstein, 1994b), relaxes into a looser arrangement (Tepass and Hartenstein, 1995). An important question is how the molecular pathways controlling migration and those controlling MET are related. There is clearly significant overlap since some genes such as Laminins are required for both the MET (Yarnitzky and Volk, 1995) and migration (Urbano et al., 2009). However, the pathways cannot be exactly the same since the relative strength of migration phenotypes versus MET phenotypes is different for different genes. For, example Integrin *βPS^−^* mutants, which have delayed midgut migration, still show basal localisation of Filamin-1 (Devenport and Brown, 2004) while migration rates appear normal in *shg* mutants, but the MET is disrupted (Tepass and Hartenstein, 1994b). Similarly, in this work, we have found that loss of *fra* most strongly affected migration, while loss of netrins had a stronger affect on MET.

While this could indicate separate molecular pathways, an alternative explanation is suggested by recent work in the worm showing similar, differential phenotypes in *unc-6* and *unc-40* mutants (Wang et al., 2014). In *unc-6* mutants, clusters of UNC-40 and FActin are not reduced in intensity but are no longer polarised to the basal side, whereas in *unc-40* mutants accumulations of FActin are reduced, but still polarise to the basal surface. Thus, in the absence of UNC-6, UNC-40 can cluster and promote FActin, but stabilization of those clusters on the basal side requires UNC-6. Similarly, in our system, Netrins were not needed for FActin accumulations and protrusions, but were essential to polarise Fra, FActin and Filamin-1 to the basal side, and this was clearly crucial for the formation of an epithelium. Thus, while Fra clearly plays a role in the MET, the role of Netrin in localising Fra to the basal side appears to be even more important.

### Netrin and Fra are endocytosed in the midgut cells

Our results also indicate that Netrins and Fra undergo mutually dependent endocytosis on the basal side of midgut cells. NetB and Fra both colocalised with the early endosome marker Rab5, and inhibition of the early endocytic pathway reduced the number of both NetB and Fra puncta. Furthermore, ligand and receptor internalisations were mutually dependent upon each other since Fra puncta were lost in *netAB^Δ^* mutants and NetB puncta were lost in *fra* mutants. NetB and FraHA also showed substantial colocalisation (i.e. ∼40%) with each other, and, given that only NetB was being detected, the total proportion of FraHA/Netrin-positive vesicles could be greater. Fra also colocalised with the late endosome marker Rab7 suggesting that it is degraded in the lysosome, which raises the question of whether downregulation of Fra might be necessary for the MET to occur.

In axon guidance, Netrin binding to DCC in the growth cone leads to rapid endocytosis and degradation of DCC, which is thought to desensitise the growth cone, allowing it to adapt to increasing basal levels of a ligand as it moves up a concentration gradient (Piper et al., 2005). Adaptation to a gradient seems unlikely in the midgut, however, since all VM cells appear to express NetB at similar levels meaning that the concentration of NetB along the length of VM should be relatively constant. Nevertheless, removal of Fra from the membrane in midgut cells might be required to attenuate the Netrin-dependent activation of motility pathways, so that a transition to an epithelial cell type can occur.

That said, we were not able to detect any disruption to either migration or the MET when endocytosis was inhibited. However, since endocytic pathways are known to be crucial for other cell migration events (reviewed in Jones et al., 2006; Le Roy and Wrana, 2005) including those that are Integrin-dependent (Caswell and Norman, 2008) we expect that the lack of a phenotype is because the time frame over which the migration/MET event takes place (∼1–2 h) is too short for the down-stream consequences of inhibiting endocytosis by Rab5^DN^ to manifest. To definitively test the importance of Fra degradation on the MET it will be necessary to find ways of specifically disrupting Fra trafficking, either by mutations to *fra* or by finding other proteins that regulate Fra but do not disrupt endocytosis in general.

### Interactions between Netrins and Integrins

Given both the Netrin/Fra pathway and the Integrin pathway regulate early midgut development an important question now is: how do these two pathways interact? Our data showing that combined loss of Fra and βPS has a clear additive phenotype indicates that, to a large degree, the pathways act in parallel. Nevertheless there are several hints that cross talk may exist. Firstly, the migration phenotype of *fra* mutants was slightly enhanced by loss of netrins. While this could potentially be due to very low levels of maternal Fra persisting in *fra* mutants, which we are not able to detect, another possibility is that Netrins are directly interacting with Integrins, as has been demonstrated in vertebrate studies (Yebra et al., 2003).

However, we also found that mutations to both netrins and *fra* had a subtle effect on Integrin localisation, in that the basal gradient of βPS within PMECs was strongly reduced, and an increased signal of both βPS and αPS1 was detected at the PMEC/VM interface. This phenotype was also seen when endocytosis was inhibited suggesting that Fra signaling may regulate turnover of the βPS/αPS1 Integrin. One possible mechanism for this is through shared downstream pathway components of Fra/DCC family receptors and Integrins (reviewed in Nikolopoulos and Giancotti, 2005). For example, activation of both Integrins and DCC receptors can lead to activation of FAK and Src family kinases (SFKs) (reviewed in Huttenlocher and Horwitz, 2011; Ren et al., 2004; Stein et al., 2001; Sun et al., 2011). Similarly, RhoGTPases, such as Rac and Cdc42, act downstream of both Integrins (reviewed in DeMali et al., 2003; Pirraglia et al., 2013; Price et al., 1998; Yu et al., 2005) and DCC (Li et al., 2002; Shekarabi and Kennedy, 2002). Thus, activation of Fra could potentially increase the pools of signaling factors in basal parts of the midgut cells, which could then promote Integrin turnover.

Whether *Fak56* or SFKs could mediate this effect is not clear, however. *Fak56* mutants have no obvious effect on midgut migration or development (Grabbe et al., 2004), and turnover of Integrin complexes in myotendinous junctions was not affected by FAK disruption (Yuan et al., 2010). Also, a Fra transgene in which all Tyr residues in the cytoplasmic domain are mutated to Phe can fully rescue *fra* midline crossing defects (O'Donnell and Bashaw, 2013) suggesting Fra phosphorylation by Src or other Tyrosine kinases may not be important in *Drosophila*. However, *Fak56* and SFKs do appear to act downstream of αPS3/βν in regulating neuromuscular junction growth (Tsai et al., 2008) and this Integrin heterodimer is expressed in the midgut (Devenport and Brown, 2004), so further investigation is warranted. In the case of the Rho GTPases, both Rac1 and Cdc42 have been implicated in midgut migration (Martin-Bermudo et al., 1999), and loss of the Rac GEF *trio* enhances *fra* midline phenotypes (Forsthoefel et al., 2005) so again it will be important to determine whether disruption of these signaling components affects Integrin localisation.

In summary, our results provide a new model for MET in which migrating cells are guided along a pathway by a chemoattractant, but subsequently become apico-basally polarised in response to that same chemoattractant, and are thereby induced to form an epithelium. It will be important now to map out the pathways acting downstream of Fra and see how these might interact with Integrins, as well as establishing the mechanism, and importance of Fra endocytic trafficking in the MET.

## Supplementary Material

Supplementary Material
