# Supplementary material for: Netrins and Frazzled/DCC promote the migration and mesenchymal to epithelial transition of Drosophila midgut cells
Source: Biol Open. 2015 Jan 23;4(2):233–43. doi: 10.1242/bio.201410827 (PMC4365492; doi:10.1242/bio.201410827)
Supplement: Supplementary Material [file supp_4_2_233__index.html]

Netrins and Frazzled/DCC promote the migration and mesenchymal to epithelial transition of Drosophila midgut cells — Supplementary Material 

# Netrins and Frazzled/DCC promote the migration and mesenchymal to epithelial transition of *Drosophila* midgut cells

## bio.201410827 Supplementary Material

**Files in this Data Supplement:**

- Supplementary Material - Melissa Pert et al. doi: 10.1242/bio.201410827
- Movie 1 - **3D rendering of a stage 13 *w1118* embryo stained for the cell adhesion molecule Fas2 to highlight the cell morphology and arrangement.** A columnar epithelium has formed.
- Movie 2 - **3D rendering of a stage 13 *netABΔ* mutant embryo stained for the cell adhesion molecule Fas2 to highlight the cell morphology and arrangement.** A columnar epithelium has not formed: cells are more rounded and disordered.
