# Supplementary material for: Netrins and Frazzled/DCC promote the migration and mesenchymal to epithelial transition of Drosophila midgut cells
Source: Biol Open. 2015 Jan 23;4(2):233–43. doi: 10.1242/bio.201410827 (PMC4365492; doi:10.1242/bio.201410827)
Supplement: Supplementary Material [file supp_bio.201410827_bio.201510827-s1.pdf]

Supplementary Material
Melissa Pert et al. doi: 10.1242/bio.201410827

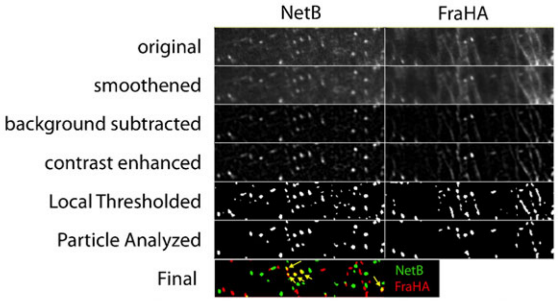

Fig. S1. Quantification of NetB/FraHA colocalisation. NetB and FraHA colocalisation was performed in stage 13 embryos using an image-processing pipeline in ImageJ. For each embryo, 10 z-slices, with a z-step size of 2 microns, were analysed for vesicles as follows. Each channel was independently i) smoothened; ii) background-subtracted (using the rolling ball method with radius=3); iii) contrast-enhanced (0.1% saturated pixels); iv) locally thresholded (using Mean method, radius=5, constant=−30; and finally put through an ''Analyze Particles'' pass to exclude puncta that were too small (A<8pixels), or which had an elongated shape (circularity<0.6) such as regions of FraHA expression along the plasma membrane. A typical region with processing steps is shown. For each embryo, the total number (combined value from the 10 slices) of vesicles was determined, which were positive for FraHA&NetB, FraHA alone or NetB alone (yellow arrows depict colocalisation).

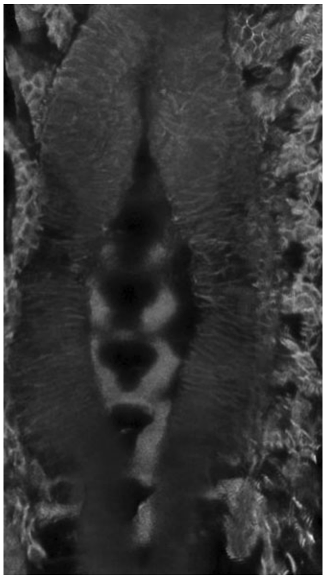

Movie 1. 3D rendering of a stage 13 w^{1118} embryo stained for the cell adhesion molecule Fas2 to highlight the cell morphology and arrangement. A columnar epithelium has formed.

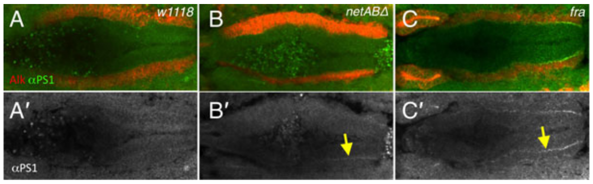

Fig. S2. Netrin and Fra regulate αPS1 localisation. (A–C) αPS1 is not obviously localised in w^{1118} control embryos (n=12) (A′) but a faint line at the midgut/VM interface is seen in *netAB*$^{\Delta}$ embryos (n=10/13) (B′, arrow) and this is even clearer in *fra*^{3}/*Df(2R)BSC880* mutants (n=5/5) (C′, arrow).

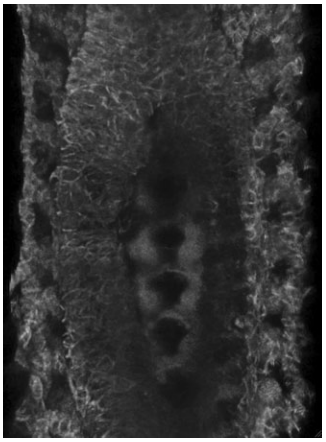

Movie 2. 3D rendering of a stage 13 *netAB*$^{\Delta}$ mutant embryo stained for the cell adhesion molecule Fas2 to highlight the cell morphology and arrangement. A columnar epithelium has not formed: cells are more rounded and disordered.
